# Supplementary material for: Identifying patterns in amyotrophic lateral sclerosis progression from sparse longitudinal data
Source: Nat Comput Sci. 2022 Sep 8;2(9):605–16. doi: 10.1038/s43588-022-00299-w (PMC10766562; doi:10.1038/s43588-022-00299-w)
Supplement: Supplementary file 1 — Supplementary Tables 1–10, Figs. 1–8, Algorithm 1, notes and Acknowledgements. [file 43588_2022_299_MOESM1_ESM.pdf]

nature portfolio

https://doi.org/10.1038/s43588-022-00299-w

Supplementary information

Identifying patterns in amyotrophic lateral sclerosis progression from sparse longitudinal data

In the format provided by the authors and unedited

Supplementary Information

Ramamoorthy et al., Identifying Patterns of ALS Progression from Sparse Longitudinal Data

Table of Contents

Supplementary Tables **3**

Supplementary Figures **7**

Supplementary Notes **14**

Modeling Approach *14*

Gaussian Process Regression 14

Dirichlet Process Clustering 14

Monotonic Inductive Bias 15

Baseline Model 16

Model Workflow 18

Study Populations *18*

Supplementary Acknowledgements **19**

Supplementary Tables Index

Supplementary Table 1. Study Populations 3
Supplementary Table 2. Study Populations – Extended Summary Statistics 3
Supplementary Table 3. Percentage of patients that have a reduction in error when MoGP is used as compared to LKM 4
Supplementary Table 4. Percentage of patients that have a reduction in error when MoGP is used as compared to per-patient slope model (SM) 4
Supplementary Table 5. Percentage of patients that have a reduction in error when MoGP is used as compared to per-patient sigmoidal model (SG) 4
Supplementary Table 6. Number of clusters in each of the models, across study populations 5
Supplementary Table 7. Study population summary statistics for participants included in prediction (≥ 4 visits) and interpolation (≥ 10 visits) experiments 5
Supplementary Table 8. Summary statistics for dominant ALS progression patterns 5
Supplementary Table 9. Frequency of MCI to AD conversions, for each ADNI cluster 6
Supplementary Table 10. Percentage of individuals in dominant PD patterns with a stable PIGD or TD diagnosis 6

Supplementary Figures Index

Supplementary Figure 1. Number of clusters in MoGP and LKM models for interpolation and prediction tests, across PRO-ACT and CEFT datasets 7
Supplementary Figure 2. Parameter sensitivity analysis showing effect of scaling alpha on the prediction experiments for CEFT, both for relative error and number of clusters 8
Supplementary Figure 3. Comparing model performance for interpolation and prediction against additional patient-specific baseline models 9
Supplementary Figure 4. Prediction and interpolation results on additional datasets 10
Supplementary Figure 5. Clusters spanning 90% of all individuals in ADNI 11
Supplementary Figure 6. Clusters spanning 90% of all individuals in PPMI 12
Supplementary Figure 7. Dominant AD progression patterns, using length-scale and mean function slope 13
Supplementary Figure 8. Dominant PD progression patterns, using length-scale and mean function slope 13

Supplementary Algorithms Index

Supplementary Algorithm 1. Mixture of Gaussian Processes Algorithm 18

Supplementary Tables

Dataset	Study Type	Total No. Participants Included	Median (IQR) No. Visits	Median (IQR) Months Followed	Median (IQR) ALSFRS-R Slope
PRO-ACT	Clinical Trial	2923	9 (7)	11.95 (4.63)	-0.67 (0.64)
NATHIST	Observational	907	5 (3)	17.24 (19.56)	-0.65 (0.62)
CEFT	Clinical Trial	476	9 (7)	16.80 (14.40)	-0.84 (0.63)
AALS	Observational	456	5 (3)	13.79 (10.55)	-0.55 (0.62)
EMORY	Observational	399	4 (3)	15.04 (13.76)	-0.89 (0.67)

Supplementary Table 1. Study Populations
Abbreviations: PRO-ACT = Pooled Resource Open-Access ALS Clinical Trials; NATHIST= ALS/MND Natural History Consortium database; CEFT = Clinical Trial Ceftriaxone in Subjects With ALS; AALS = Answer ALS; EMORY = Emory ALS Clinic. IQR indicates interquartile range. Slope (points per month) is calculated as an anchored linear regression across all data points, with the anchor indicating an imputed value of 48 at symptom onset.

Dataset	No. (%) Male	No. (%) Female	No. (%) Limb Onset	No. (%) Bulbar Onset	Median (IQR) Age of Onset	Total No. Participants Included
PRO-ACT	1838 (62.9)	1085 (37.1)	2001 (68.5)	589 (20.2)	55.10 (16.11)	2923
NATHIST	537 (59.2)	368 (40.6)	621 (68.5)	239 (26.4)	61.73 (14.24)	907
CEFT	289 (60.7)	187 (39.3)	377 (79.2)	108 (22.7)	54.70 (15.12)	476
AALS	287 (62.9)	169 (37.1)	344 (75.4)	111 (24.3)	57.85 (13.96)	456
EMORY	233 (58.4)	166 (41.6)	N/A	N/A	61.09 (16.40)	399

Supplementary Table 2. Study Populations – Extended Summary Statistics
IQR indicates interquartile range. N/A indicates not reported.

	RMSE Difference (ALSFRS-R points)					
**Dataset**	> 0	> 1	> 2	> 3	> 4	> 5
**AALS**	71.27%	27.19%	8.33%	2.85%	0.88%	0.22%
**CEFT**	75.42%	39.29%	16.18%	6.51%	3.78%	1.26%
**EMORY**	74.44%	31.08%	11.78%	5.01%	2.01%	1.50%
**NATHIST**	75.96%	37.71%	14.33%	6.73%	3.09%	1.43%
**PRO-ACT**	77.87%	27.16%	9.99%	3.73%	1.27%	0.31%

Supplementary Table 3. Percentage of patients that have a reduction in error when MoGP is used as compared to LKM

ALSFRS-R point thresholds range from 0 ALSFRS-R points (indicates percent of all participants for whom a MoGP provides predictions with a lower error than LKM) to 5 ALSFRS-R points (percentage of patients that have a reduced error of more than 5 ALSFRS-R points using a MoGP).

	RMSE Difference (ALSFRS-R points)					
**Dataset**	> 0	> 1	> 2	> 3	> 4	> 5
**AALS**	37.28%	11.40%	3.29%	1.32%	0.44%	0.00%
**CEFT**	59.24%	24.37%	6.30%	2.73%	2.10%	0.42%
**EMORY**	45.61%	11.03%	4.76%	2.51%	1.50%	0.75%
**NATHIST**	54.47%	18.74%	6.73%	3.09%	1.43%	0.66%
**PRO-ACT**	61.27%	19.43%	5.71%	1.95%	0.58%	0.17%

Supplementary Table 4. Percentage of patients that have a reduction in error when MoGP is used as compared to per-patient slope model (SM)

	RMSE Difference (ALSFRS-R points)					
**Dataset**	> 0	> 1	> 2	> 3	> 4	> 5
**AALS**	41.01%	9.43%	3.51%	0.66%	0.22%	0.00%
**CEFT**	32.14%	4.20%	0.21%	0.00%	0.00%	0.00%
**EMORY**	35.59%	9.02%	3.01%	1.25%	0.75%	0.50%
**NATHIST**	41.57%	12.13%	3.09%	0.77%	0.22%	0.22%
**PRO-ACT**	45.54%	4.21%	0.27%	0.00%	0.00%	0.00%

Supplementary Table 5. Percentage of patients that have a reduction in error when MoGP is used as compared to per-patient sigmoidal model (SG)

Dataset	No. RBF clusters	No. LKM clusters	No. slope models
**AALS**	22	25	456
**CEFT**	34	44	476
**EMORY**	25	30	399
**PRO-ACT**	92	127	2923
**NATHIST**	41	60	907

Supplementary Table 6. Number of clusters in each of the models, across study populations

Because a slope model was fit to each patient, the number of slope models is equivalent to number of patients included in the training data.

Dataset	Inclusion Criteria	Total No. Participants Included	Median (IQR) No. Visits	Median (IQR) Months Followed	Median (IQR) ALSFRS-R Slope
PRO-ACT	≥ 4 visits	2814	9 (8)	11.95 (4.43)	-0.66 (0.63)
CEFT	≥ 4 visits	453	10 (7)	18.00 (13.20)	-0.81 (0.59)
NATHIST	≥ 4 visits	714	6 (3)	20.00 (20.66)	-0.61 (0.62)
AALS	≥ 4 visits	341	5 (2)	15.86 (11.49)	-0.49 (0.57)
EMORY	≥ 4 visits	283	5 (3)	18.95 (14.94)	-0.86 (0.62)
PRO-ACT	≥ 10 visits	1327	14 (5)	13.46 (3.97)	-0.66 (0.58)
CEFT	≥ 10 visits	228	13 (5)	25.20 (10.80)	-0.69 (0.46)
NATHIST	≥ 10 visits	132	12 (6)	44.76 (24.52)	-0.42 (0.33)

Supplementary Table 7. Study population summary statistics for participants included in prediction (≥ 4 visits) and interpolation (≥ 10 visits) experiments

IQR indicates interquartile range. Slope (points per month) is calculated as an anchored linear regression across all data points, with the anchor indicating an imputed value of 48 at symptom onset.

Pattern	Median Age of Onset	Limb Onset Frequency	Limb Onset P-Val	Bulbar Onset Frequency	Bulbar P-Val
a	59.09	66.67% (20)	1.50E-01	13.33% (4)	1.30E-01
b	57.18	58.51% (299)	4.63E-08	30.14% (154)	8.73E-10
c	58.45	55.56% (5)	1.88E-01	22.22% (2)	3.03E-01
d	54.88	65.48% (590)	2.41E-03	20.64% (186)	3.59E-02
e	54.16	76.97% (391)	9.28E-07	14.37% (73)	5.95E-05
f	54.17	72.20% (696)	3.07E-04	17.63% (170)	2.26E-03

Supplementary Table 8. Summary statistics for dominant ALS progression patterns

Patterns shown in Extended Data Figure 7. P-Values calculated with hypergeometric test.

Cluster	Cluster Size	% (No.) Baseline AD	% (No.) Baseline MCI	AD p-val	MCI p-val
A	59	47.46% (28)	52.54% (31)	1.13E-01	1.14E-01
B	49	61.22% (30)	38.78% (19)	1.04E-02	1.40E-02
C	30	16.67% (5)	80.00% (24)	3.04E-04	8.95E-04
D	26	88.46% (23)	11.54% (3)	4.32E-06	6.62E-06
E	23	17.39% (4)	82.61% (19)	2.19E-03	1.68E-03
F	20	10.00% (2)	90.00% (18)	3.84E-04	2.88E-04
G	19	94.74% (18)	5.26% (1)	6.39E-06	9.07E-06
H	17	23.53% (4)	76.47% (13)	3.03E-02	2.60E-02

Supplementary Table 9. Frequency of MCI to AD conversions, for each ADNI cluster
Clusters shown in Extended Data Figure 8. Percent baseline Alzheimer's Disease (AD) or Mild Cognitive Impairment (MCI) diagnosis calculated for each cluster. P-values calculated using hypergeometric test.

Pattern	% (No.) TD	% (No.) PIGD	TD P-val	PIGD P-val
a	N/A	N/A	N/A	N/A
b	75.00% (3)	25.00% (1)	4.19E-01	4.19E-01
c	N/A	N/A	N/A	N/A
d	81.25% (13)	18.75% (3)	2.82E-01	2.82E-01
e	45.45% (5)	54.55% (6)	3.89E-03	3.89E-03
f	91.89% (34)	8.11% (3)	1.10E-02	1.10E-02

Supplementary Table 10. Percentage of individuals in dominant PD patterns with a stable PIGD or TD diagnosis
Dominant patterns shown in Supplementary Figure 8. N/A indicates none of the individuals had a stable postural instability/gait difficulty (PIGD) or tremor dominant (TD) label within that cluster.

Supplementary Figures

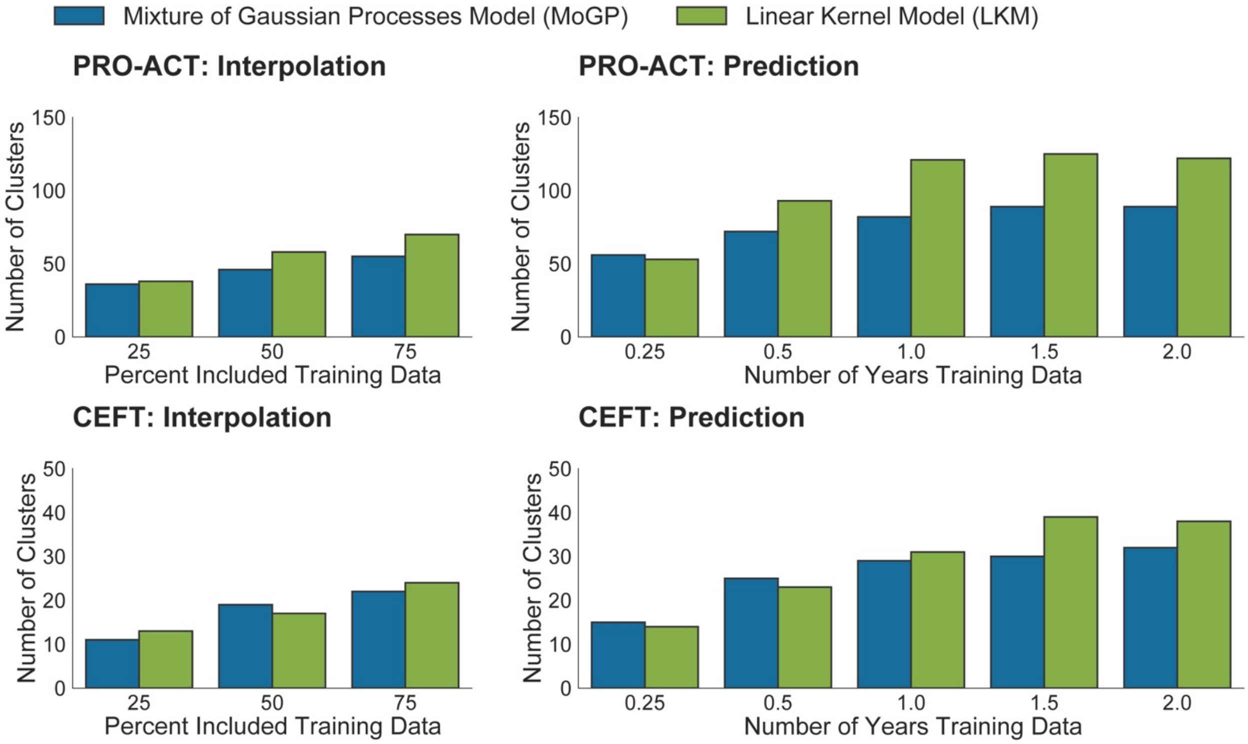

Supplementary Figure 1. Number of clusters in MoGP and LKM models for interpolation and prediction tests, across PRO-ACT and CEFT datasets

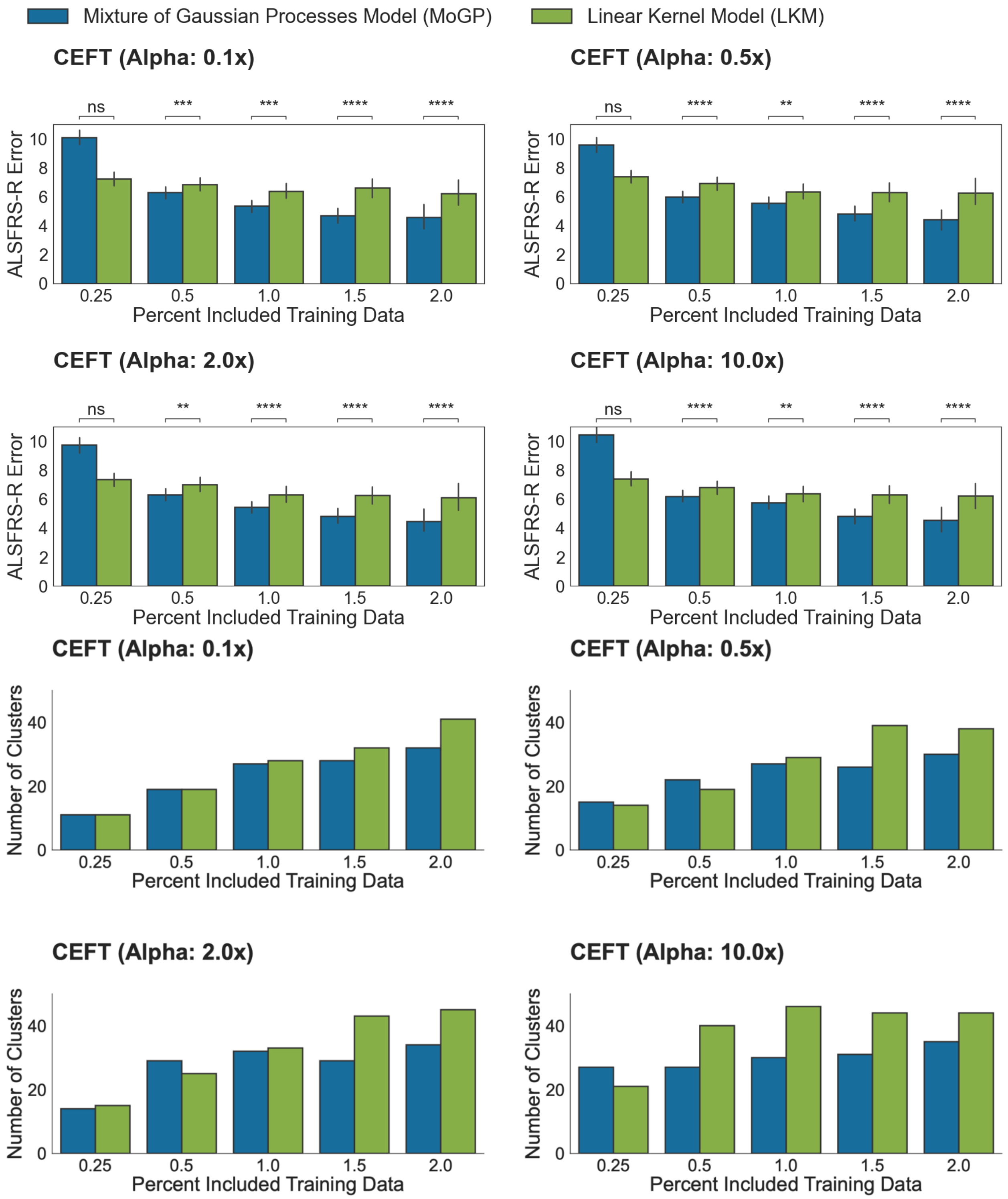

Supplementary Figure 2. Parameter sensitivity analysis showing effect of scaling alpha on the prediction experiments for CEFT, both for relative error and number of clusters

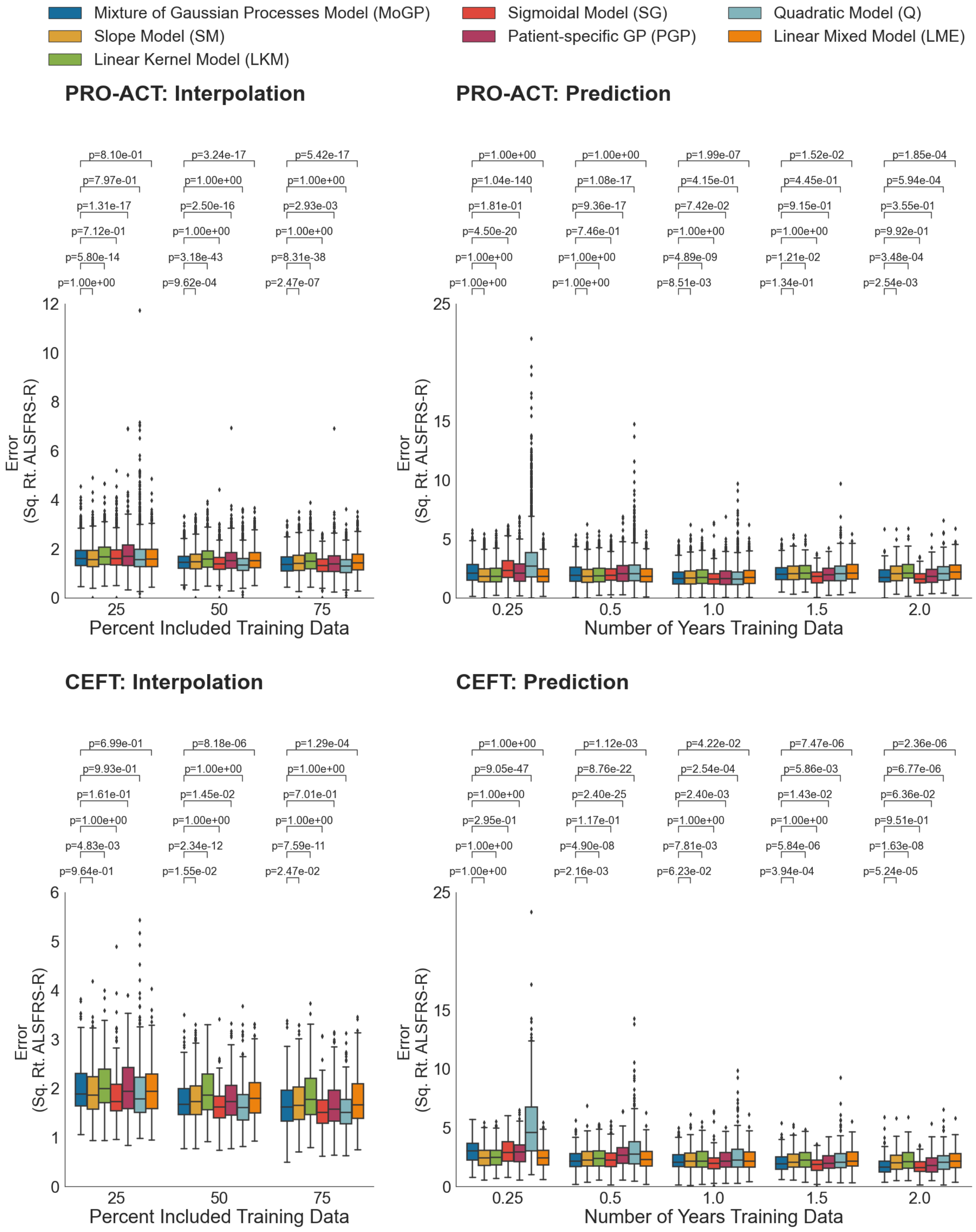

Supplementary Figure 3. Comparing model performance for interpolation and prediction against additional patient-specific baseline models

P-values calculated with Wilcoxon signed-rank one-sided test. Box plot represents interquartile range around mean; whiskers indicate proportion (1.5) of the IQR past the low and high quartiles to extend the plot whiskers. Points outside the whisker range represent outlier samples.

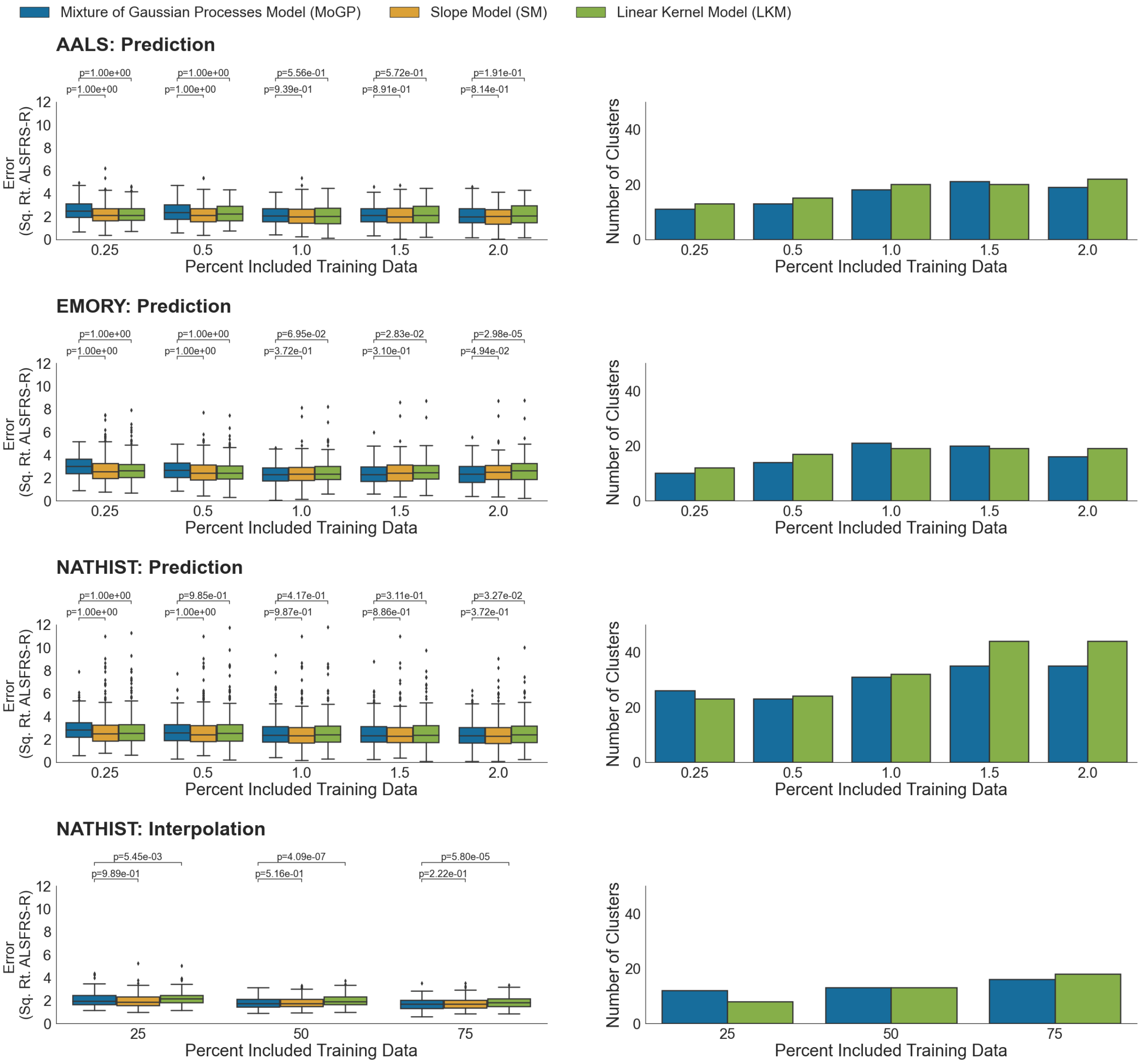

Supplementary Figure 4. Prediction and interpolation results on additional datasets
P-values calculated with Wilcoxon signed-rank one-sided test. Box plot represents interquartile range around mean; whiskers indicate proportion (1.5) of the IQR past the low and high quartiles to extend the plot whiskers. Points outside the whisker range represent outlier samples.

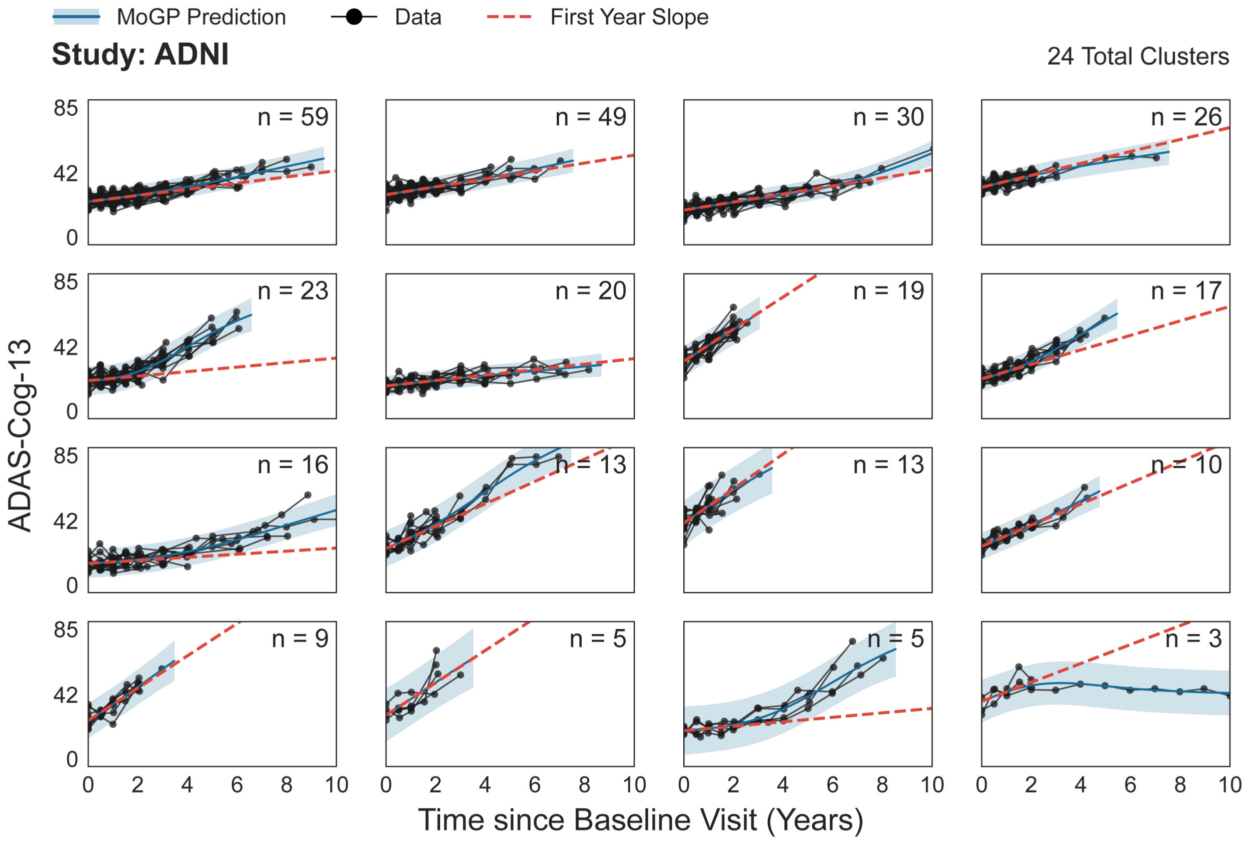

Supplementary Figure 5. Clusters spanning 90% of all individuals in ADNI
The first year slope is calculated as: (mean cluster at one year after first visit – mean cluster score at first visit value), divided by the time between the two. N indicates the number of individuals in each cluster.

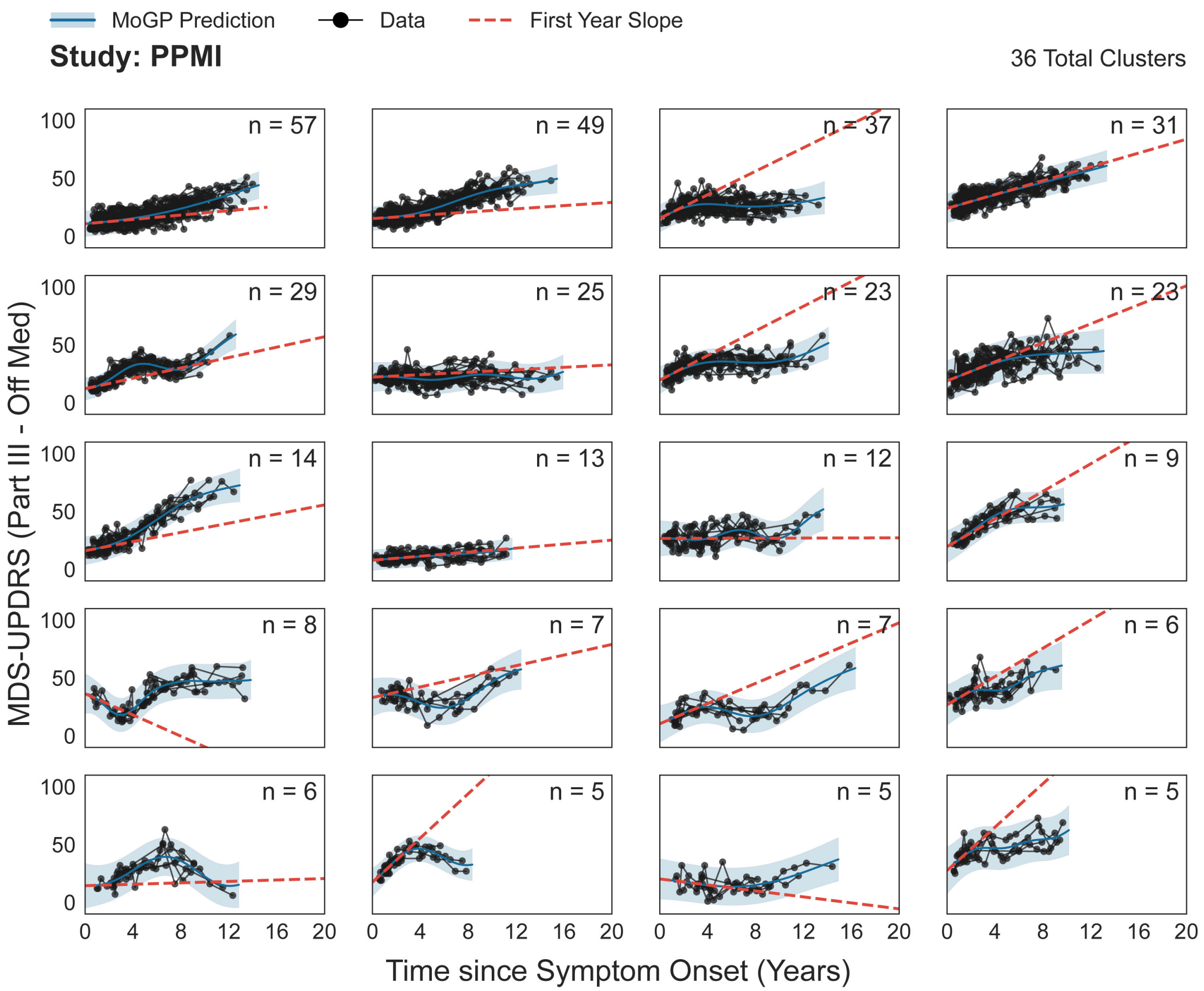

Supplementary Figure 6. Clusters spanning 90% of all individuals in PPMI

The first year slope is calculated as: (mean cluster at one year after symptom onset – mean cluster score at symptom. onset), divided by the time between the two. N indicates the number of individuals in each cluster.

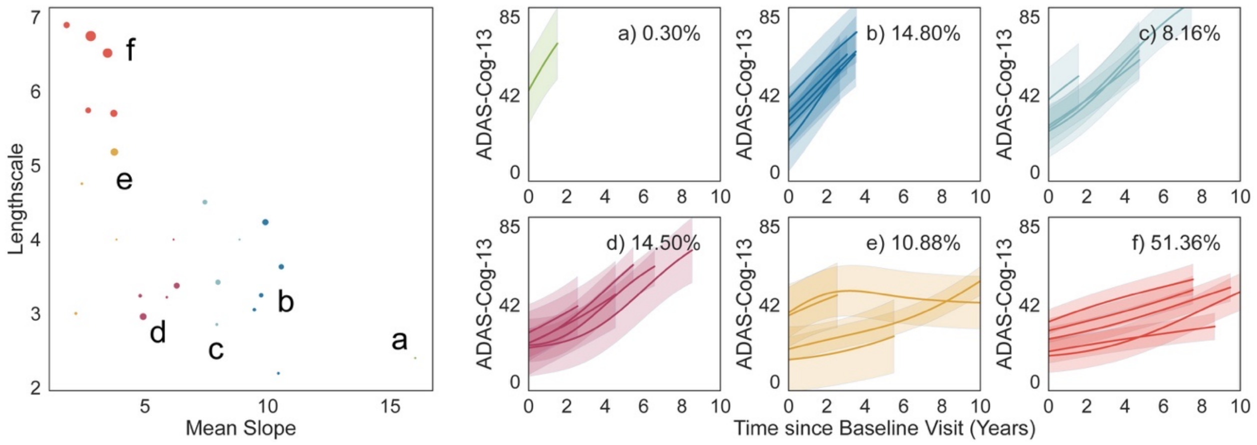

Supplementary Figure 7. Dominant AD progression patterns, using length-scale and mean function slope

Length-scale indicates trajectory stability; mean function slope corresponds to rate of progression. Learned model parameters are k-means clustered (Left plot; k=6, marker size corresponds to cluster size), with clusters ≥ N=1 visualized, and percentage of individuals that fall within each of the trajectory patterns labeled (Right plots).

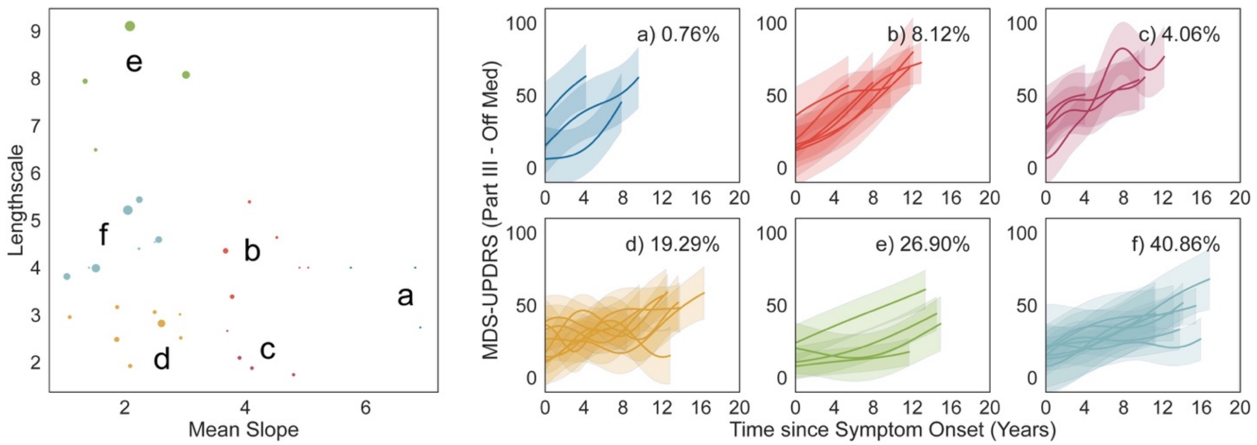

Supplementary Figure 8. Dominant PD progression patterns, using length-scale and mean function slope

Length-scale indicates trajectory stability; mean function slope corresponds to rate of progression. Learned model parameters are k-means clustered (Left plot; k=6, marker size corresponds to cluster size), with clusters ≥ N=1 visualized, and percentage of individuals that fall within each of the trajectory patterns labeled (Right plots).

Supplementary Notes

Modeling Approach

Gaussian Process Regression
Gaussian processes take the form:

$$f(x) \sim GP\ (m(x), k(x, x'))$$

where m(x) describes the model's mean function and k(x,x') describes the model's covariance function. To specify the covariance function, the Gaussian processes in this implementation of MoGP uses the SE kernel, with the form:

$$k(x, x') = \sigma^2 exp\ (-\frac{(x - x')^2}{2l^2})$$

where σ^2 is the signal variance and l is the length-scale. The signal variance (σ^2) determines the average distance of the function from the mean. The length-scale (l) specifies the smoothness of the function, with increasing length-scales resulting in smoother functions. For the length-scale, a gamma prior with a mean of 4 and variance of 9 was used. The length-scale prior is approximately half of the maximum trajectory duration included in our model, selected to encourage minimal mean function crossings.

In contrast to the SE kernel, the LKM kernel is a linear kernel added to a bias kernel, with the form:

$$k(x, x') = \sigma_v^2 (x)(x') + \sigma_b^2$$

where σ_v^2 and σ_b^2 are the signal and bias variance, respectively. The bias allows for a non-zero intercept.

For both Gaussian Process kernels, a fixed signal variance of 1 was used to train the models. A gamma prior with mean 0.75 and variance of 0.25^2 was used for the likelihood noise variance, selected to account for noise present in the data; this parameter was optimized through model training.

Dirichlet Process Clustering
This implementation of Dirichlet Process (DP) clustering uses a collapsed Gibbs sampler, in which we iteratively assign probabilities of each sample joining either existing clusters or generating a new cluster to calculate the likelihood of cluster

membership. By repeating this process for each sample until convergence, we are able to identify the number of clusters the data captures as well as sample-specific cluster assignments.

The DP clustering model in MoGP takes the form:

$$G(f) = \sum_{k=1}^{\infty} \pi_k \delta_{f_k}(f)$$

where f indicates a cluster-specific GP regression function, k indicates cluster membership, and π_k indicates cluster-specific probability, where:

$$\pi_k = \beta_k \prod_{j=1}^{k-1}(1 - \beta_j) \text{ and } \beta_k \sim Beta\,(\cdot\,|1, \propto)$$

$\propto$ indicates the scaling parameter and modifying this can influence the degree of cluster discretization and therefore the number of identified clusters. For these experiments, to encourage large clusters with at least 50 individuals per cluster, alpha was set to the number of patients in a given dataset divided by 50.

We also show a parameter sensitivity analysis of the prediction experiment to varying alpha values (Supplementary Figure 2). Four different alpha values were tested: 0.1, 0.5, 2.0, and 5.0 times the original mixing parameter. Both the overall error as well as the number of clusters is reasonably stable across the experiments. In the absence of structure in the data, we would expect the mixing parameter to have a direct effect on the cluster sizes; however, the stability of the results across these parameters points to data structure that is learned in the training process.

The results instead indicate that overfitting likely drives the differences in the model performance. The experiments with fewer training data points are more susceptible to overfitting in the case of a complex model like the flexible gaussian process; however, as more data is provided, the MoGP better captures the data structure.

Monotonic Inductive Bias

To encourage monotonically declining functions, we use two modifications to MoGP: 1) a negative linear mean function in our GPs, and 2) a thresholding function to determine cluster membership. In our sampling procedure for our DP model, the probability of each individual joining each cluster is calculated. Our thresholding function constrains the number of clusters an individual can join. If the score for initial visit for a given sample is not close (where close is defined by a user-set ‘threshold’ parameter) to a cluster’s mean function, then the algorithm sets the probability of joining that cluster to

0. This prevents the probability that a participant with a starting ALSFRS-R score vastly divergent from a given cluster will be added to the cluster. For these experiments, this threshold is set as 0.5 for z-scored data, which roughly approximates 5 ALSFRS-R points, because it would be clinically unlikely that one sees this large of a range in patient function for a trajectory pattern. For our negative linear mean function, we used a gamma prior with a mean of 0.66 and variance of 0.2. Together, these values were chosen to minimize major deviation from a monotonic trajectory, although these are weak priors that allow for a degree of non-monotonic behavior depending on the data.

One limitation for our monotonic inductive bias is that our priors are relatively weak. This means that occasionally, outliers or variance in data may cause non-monotonic behavior in some clusters, particularly those that are small; an example of this can be seen in one of the clusters in Extended Data Figure 2. Future work can involve strengthening this constraint by modifying the optimization of the GP kernel.

Baseline Model

Since there are many settings in which patient-specific parametric models are very useful, we provide additional characterization of per-patient parametric models using our framework: a personalized-GP, the D50 sigmoidal model[20,21], a quadratic model, and a linear mixed effect model.

The personalized-GP model is initialized with the same priors as our MoGP model, and optimized using GPy (see Supplementary Algorithm 1 for GP model priors).

Our D50 model is implemented in the following form:

$$y = \frac{48}{1 + e^{\frac{(x - D50)}{dx}}}$$

where D50 = time point when the ALSFRS-R drops to 24; dx = slope of ALSFRS-R decrease. The model parameters are fit using scipy curve_fit (dogbox method, with bounds ((0.1, 0.1), (75, 5)), with a D50 initial value of 5, and a dx initial value of 0.5.

Our quadratic model is implemented in the following form:

$$y = ax^2 + bx + c$$

where coefficients a, b, and c are fit using scipy curve fit (dogbox method, initial values a=1, b=1, c=1, no bounds).

Our linear mixed model is implemented using statsmodels.formula.api.mixedlm, with the design “Y~x”, and groups indicating individual patients. The model is fit using the lbgfs method. In some cases of sparse data, the linear mixed model did not reach convergence; in these cases, the best performance was reported.

Model Workflow
The below section details the specifications for model training, optimization, and parameter initialization.

Supplementary Algorithm 1. Mixture of Gaussian Processes Algorithm
Input: Initial number of clusters, model priors*
Output: Optimized model parameters, latent cluster probabilities

Initialize model parameters and cluster assignments

For 1, …, N iterations do

  For 1, …, N patients in random order do

    Remove patient from current cluster

    For existing clusters 1, …, K

      Compute the probability of assigning patient to cluster conditioned on the cluster assignments of all other patients, all other patient trajectories, and model priors.

      Compute the probability of assigning the patient to a new cluster conditioned on the cluster assignments of all other patients, all other patient trajectories, and model priors.

      Sample a new cluster assignment according to the computed cluster assignment probabilities.

    Add patient observation to cluster based on sample

    Remove empty clusters, if needed

***Generative model and priors**:
GP Regression:
Signal Variance: Fixed to 1
Length-scale: Gamma prior with mean 4., variance 9
Mean function slope: Gamma prior with mean 2/3, variance 0.2
Noise variance: Gamma prior with mean 0.75, variance 0.25**2
Threshold: 0.5 for z-score normalized data

Number of iterations: 100

Study Populations
Explicit approval was received for all clinical datasets used in the present work. For AALS, the study was approved by local institutional review boards, and all participants provided written informed consent. Consent was uniform across all sites and included agreement to share data broadly for medical research. We received approval for CEFT

from the National Institute of Neurological Disorders and Stroke (NINDS). For the original CEFT study, institutional review board approval was obtained at each center, as well as the MGH coordination center IRB, and participants provided written informed consent before screening. We received approval for PRO-ACT from the Pooled Resource Open-Access ALS Clinical Trials Consortium. PRO-ACT is an anonymized database that includes merged datasets from multiple ALS clinical trials. It requires an application to request access, in which the user must agree to protect the security of the data. Dr. Jonathan Glass provided approval and access for using the EMORY dataset. For the original EMORY dataset, the Emory institutional review board approved the study. For NATHIST, each individual site had local IRB approval.

Data used in the preparation of this article were obtained from the Pooled Resource Open-Access ALS Clinical Trials (PRO-ACT) Database. In 2011, Prize4Life, in collaboration with the Northeast ALS Consortium, and with funding from the ALS Therapy Alliance, formed the Pooled Resource Open-Access ALS Clinical Trials (PRO-ACT) Consortium. The data available in the PRO-ACT Database has been volunteered by PRO-ACT Consortium members.

Data used in the preparation of this manuscript were captured by the ALS/MND Natural History Consortium and obtained from NeuroBANK® patient-centric platform hosted by Neurological Clinical Research Institute at Mass General Brigham.

Supplementary Acknowledgements

Data used in the preparation of this article were obtained from the Pooled Resource Open-Access ALS Clinical Trials (PRO-ACT) Database. As such, the following organizations and individuals within the PRO-ACT Consortium contributed to the design and implementation of the PRO-ACT Database and/or provided data, but did not participate in the analysis of the data or the writing of this report:

- Neurological Clinical Research Institute, MGH
- Northeast ALS Consortium
- Novartis
- Prize4Life Israel
- Regeneron Pharmaceuticals, Inc.
- Sanofi
- Teva Pharmaceutical Industries, Ltd.
- Knopp Biosciences

Data used in the preparation of this article were obtained from the ALS/MND Natural History Consortium. As such, the following organizations and investigators within the ALS/MND Natural History Consortium contributed to the provided data, but did not participate in the analysis of the data or the writing of this manuscript:

- Henry Ford Health Systems/ Ximena Arcila londono
- Neuromuscular Omnicenter, Milan, Italy/Christian Lunetta
- Northwestern University/Senda Ajroud-Driss
- Providence ALS Center/Nicholas Olney
- Saint Louis University School of Medicine/Ghazala Hayat
- Temple University/Terry Heiman-Patterson
- University of Florida Gainesville/James Wymer
- University of Minnesota/David Walk
- Virginia Commonwealth University Health/Kelly Graham Gwathmey
- Neurological Clinical Research Institute, MGH/Alexander Sherman
- Neurological Clinical Research Institute, MGH/Kenneth Faulconer
- Neurological Clinical Research Institute, MGH/Ervin Sanani
- Neurological Clinical Research Institute, MGH/Alex Berger
- Neurological Clinical Research Institute, MGH/Julia Mirochnick

This research includes the National Institute of Neurologic Disease and Stroke's Archived Clinical Research data (Clinical Trial of Ceftriaxone in ALS, Merit Cudkowicz, Massachusetts General Hospital) received from NINDS Archived Clinical Research Datasets webpage.

Data used in the preparation of this article were obtained from the Parkinson's Progression Markers Initiative (PPMI) database (https://www.ppmi-info.org/access-data-specimens/download-data). For up-to-date information on the study, visit www.ppmi-info.org. PPMI – a public-private partnership – is funded by the Michael J. Fox Foundation for Parkinson's Research and funding partners, including:

1. 4D Pharma
2. AbbVie Inc.
3. AcureX Therapeutics
4. Allergan
5. Amathus Therapeutics
6. Aligning Science Across Parkinson's (ASAP)
7. Avid Radiopharmaceuticals
8. Bial Biotech
9. Biogen
10. BioLegend
11. Bristol Myers Squibb

12. Calico Life Sciences LLC
13. Celgene Corporation
14. DaCapo Brainscience
15. Denali Therapeutics
16. The Edmond J. Safra Foundation
17. Eli Lilly and Company
18. GE Healthcare
19. GlaxoSmithKline
20. Golub Capital
21. Handl Therapeutics
22. Insitro
23. Janssen Pharmaceuticals
24. Lundbeck
25. Merck & Co., Inc.
26. Meso Scale Diagnostics, LLC
27. Neurocrine Biosciences
28. Pfizer Inc.
29. Piramal Imaging
30. Prevail Therapeutics
31. F. Hoffmann-La Roche Ltd and its affiliated company Genentech Inc.
32. Sanofi Genzyme
33. Servier
34. Takeda Pharmaceutical Company
35. Teva Neuroscience, Inc.
36. UCB
37. Vanqua Bio
38. Verily Life Sciences
39. Voyager Therapeutics, Inc.
40. Yumanity Therapeutics, Inc.

Data used in the preparation of this article were obtained from the Alzheimer's Disease Neuroimaging Initiative (ADNI) database (adni.loni.usc.edu). The ADNI was launched in 2003 as a public-private partnership, led by Principal Investigator Michael W. Weiner, MD. The primary goal of ADNI has been to test whether serial magnetic resonance imaging (MRI), positron emission tomography (PET), other biological markers, and clinical and neuropsychological assessment can be combined to measure the progression of mild cognitive impairment (MCI) and early Alzheimer's disease (AD). For up-to-date information, see www.adni-info.org. As such, the investigators within the ADNI contributed to the design and implementation of ADNI and/or provided data but did not participate in analysis or writing of this report. A complete listing of ADNI

investigators can be found at: http://adni.loni.usc.edu/wp-content/uploads/how_to_apply/ADNI_Acknowledgement_List.pdf. Data collection and sharing for this project was funded by the Alzheimer's Disease Neuroimaging Initiative (ADNI) (National Institutes of Health Grant U01 AG024904) and DOD ADNI (Department of Defense award number W81XWH-12-2-0012). ADNI is funded by the National Institute on Aging, the National Institute of Biomedical Imaging and Bioengineering, and through generous contributions from the following: AbbVie, Alzheimer's Association; Alzheimer's Drug Discovery Foundation; Araclon Biotech; BioClinica, Inc.; Biogen; Bristol-Myers Squibb Company; CereSpir, Inc.; Cogstate; Eisai Inc.; Elan Pharmaceuticals, Inc.; Eli Lilly and Company; EuroImmun; F. Hoffmann-La Roche Ltd and its affiliated company Genentech, Inc.; Fujirebio; GE Healthcare; IXICO Ltd.; Janssen Alzheimer Immunotherapy Research & Development, LLC.; Johnson & Johnson Pharmaceutical Research & Development LLC.; Lumosity; Lundbeck; Merck & Co., Inc.; Meso Scale Diagnostics, LLC.; NeuroRx Research; Neurotrack Technologies; Novartis Pharmaceuticals Corporation; Pfizer Inc.; Piramal Imaging; Servier; Takeda Pharmaceutical Company; and Transition Therapeutics. The Canadian Institutes of Health Research is providing funds to support ADNI clinical sites in Canada. Private sector contributions are facilitated by the Foundation for the National Institutes of Health (www.fnih.org). The grantee organization is the Northern California Institute for Research and Education, and the study is coordinated by the Alzheimer's Therapeutic Research Institute at the University of Southern California. ADNI data are disseminated by the Laboratory for Neuro Imaging at the University of Southern California.
