# Supplementary material for: Identifying patterns in amyotrophic lateral sclerosis progression from sparse longitudinal data
Source: Nat Comput Sci. 2022 Sep 8;2(9):605–16. doi: 10.1038/s43588-022-00299-w (PMC10766562; doi:10.1038/s43588-022-00299-w)
Supplement: Supplementary file 2 — Reporting Summary [file 43588_2022_299_MOESM2_ESM.pdf]

nature research

Corresponding author(s): Ernest Fraenkel

Last updated by author(s): 6/15/2022

Reporting Summary

Nature Research wishes to improve the reproducibility of the work that we publish. This form provides structure for consistency and transparency in reporting. For further information on Nature Research policies, see our Editorial Policies and the Editorial Policy Checklist.

Statistics

For all statistical analyses, confirm that the following items are present in the figure legend, table legend, main text, or Methods section.

n/a	Confirmed	
☐	☒	The exact sample size (*n*) for each experimental group/condition, given as a discrete number and unit of measurement
☐	☒	A statement on whether measurements were taken from distinct samples or whether the same sample was measured repeatedly
☐	☒	The statistical test(s) used AND whether they are one- or two-sided<br>*Only common tests should be described solely by name; describe more complex techniques in the Methods section.*
☐	☒	A description of all covariates tested
☐	☒	A description of any assumptions or corrections, such as tests of normality and adjustment for multiple comparisons
☐	☒	A full description of the statistical parameters including central tendency (e.g. means) or other basic estimates (e.g. regression coefficient) AND variation (e.g. standard deviation) or associated estimates of uncertainty (e.g. confidence intervals)
☐	☒	For null hypothesis testing, the test statistic (e.g. *F*, *t*, *r*) with confidence intervals, effect sizes, degrees of freedom and *P* value noted<br>*Give P values as exact values whenever suitable.*
☐	☒	For Bayesian analysis, information on the choice of priors and Markov chain Monte Carlo settings
☒	☐	For hierarchical and complex designs, identification of the appropriate level for tests and full reporting of outcomes
☒	☐	Estimates of effect sizes (e.g. Cohen's *d*, Pearson's *r*), indicating how they were calculated

Our web collection on statistics for biologists contains articles on many of the points above.

Software and code

Policy information about availability of computer code

Data collection	No software used to collect data.
Data analysis	We provide the python code for the MoGP framework as well as a pre-trained reference model that researchers can use to generate predictions of cluster membership and trajectory function from input patient data. We also provide a pip-installable Python package associated with this work (mogp). All code used for data processing, modeling, and figure generation can be found at: https://github.com/fraenkel-lab/mogp. Code also is deposited on Zenodo (License: BSD 3-Clause; https://doi.org/10.5281/zenodo.6744399).<br><br>Python version and specific package versions used for analysis listed below:<br>Python: 3.7.3<br>Packages:<br>joblib: 1.0.0<br>numpy: 1.19.4<br>pandas: 1.3.1<br>openpyxl: 3.0.5<br>sas7bdat: 2.2.3<br>seaborn: 0.11.1<br>statannot: 0.2.3<br>lifelines: 0.25.7<br>statsmodels: 0.12.2<br>mogp: 0.1.1<br>jupyter: 1.0.0<br>Gpy: 1.9.9<br>scipy: 1.7.3

scikit-learn: 0.21.1
sklearn: 0.0
matplotlib: 3.1.1

Computational environments: Model run on Azure and compute cluster.
Azure specifications: Standard F32s_v2 machines (32 vCPUs, 64 Gb Mem)
Cluster: 16 cores, 1 node, 10GB memory

For manuscripts utilizing custom algorithms or software that are central to the research but not yet described in published literature, software must be made available to editors and reviewers. We strongly encourage code deposition in a community repository (e.g. GitHub). See the Nature Research guidelines for submitting code & software for further information.

Data

Policy information about availability of data

All manuscripts must include a data availability statement. This statement should provide the following information, where applicable:

- Accession codes, unique identifiers, or web links for publicly available datasets
- A list of figures that have associated raw data
- A description of any restrictions on data availability

A pre-trained reference model for this study can be downloaded here: http://fraenkel.mit.edu/mogp

Source Data for Figures 2-4 and Extended Data Figure 7 are available with this manuscript. Source Data for Figure 1 and Extended Data Figure 2 are available as a Python object from http://fraenkel.mit.edu/mogp. Other source data are unavailable at this time due to containing patient-level clinical data; however, all figures can be generated using the code provided, after downloading the datasets listed below.

Clinical data for this study can be obtained from the following sources:
AALS (ClinicalTrials.gov Identifier: NCT02574390) is available for download in the AnswerALS data portal (data.answerals.org). PRO-ACT can be downloaded from the PRO-ACT database (https://nctu.partners.org/ProACT). CEFT (ClinicalTrials.gov Identifier: NCT00349622) can be downloaded from National Institute of Neurological Disorders and Stroke (NINDS) (https://www.ninds.nih.gov/Current-Research/Research-Funded-NINDS/Clinical-Research/Archived-Clinical-Research-Datasets). EMORY is restricted access at this time due to containing information that could compromise patient privacy, but available with permission from Dr. Jonathan Glass (jglas03@emory.edu) for legitimate research. Response to request will be provided within two weeks, all data provided will be fully de-identified, a DUA will need to be established, and the source data will need to be acknowledged in any publications. NATHIST is available from the ALS/MND Natural History Consortium (https://www.data4cures.org/requestingdata) with a summary of proposed data use, data elements requested, and publication intent. PPMI can be downloaded, with a data use agreement, online application, and compliance with publication policy (https://www.ppmi-info.org/access-data-specimens/download-data). Applications for data access are reviewed by the Data and Publications Committee within one week of receipt. ADNI can be downloaded through the LONI Image and Data Archive (https://adni.loni.usc.edu/data-samples/access-data/#access_data). Access is contingent on adherence to the ADNI Data Use Agreement and their publication policies. The application process includes the acceptance of a Data Use Agreement and submission of an online application form. The application must include the investigator's institutional affiliation and the proposed uses of the ADNI data. ADNI data may not be used for commercial products or redistributed in any way.

Field-specific reporting

Please select the one below that is the best fit for your research. If you are not sure, read the appropriate sections before making your selection.

☒ Life sciences ☐ Behavioural & social sciences ☐ Ecological, evolutionary & environmental sciences

For a reference copy of the document with all sections, see nature.com/documents/nr-reporting-summary-flat.pdf

Life sciences study design

All studies must disclose on these points even when the disclosure is negative.

Sample size	Analysis was conducted using only de-identfied datasets from previously collected clinical cohorts. Data for this analysis was obtained from four large study populations. Three observational ALS studies were used: Answer ALS (N=456 patients; NCT02574390) the Emory ALS Clinic database (N=399 patients), the ALS/Natural History Consortia (N=907), and two overlapping clinical trial datasets: The Pooled Resource Open-Access ALS Clinical Trials (N=2923 patients) and the Clinical Trial of Ceftriaxone in ALS (N=476; NCT00349622).
Data exclusions	Data was preprocessed prior to analysis to select participants with consistent, longitudinal data available. Participants were excluded from the model if fewer than three complete ALSFSR-R visits were recorded, the first visit was more than seven years from symptom onset, or an increase of greater than six points in ALSFRS-R between subsequent visits was recorded. These criteria were pre-established.
Replication	We conducted extensive analysis to evaluate the robustness of our results. These included comparing model results across heterogeneous clinical cohorts, withholding data and evaluating the accuracy of reconstructing that data, and using test/train sets to evaluate model transferability between datasets. Our model demonstrates strong robustness across these settings, and these results are detailed in the manuscript.
Randomization	Because our analysis does not compare a treatment and control group, randomization is not relevant to this study. For controlling for covariates: by including multiple large clinical cohorts, we were able to evaluate model performance across common sources of covariates in clinical data, such as variations in rate of progression in each cohort, frequency of clinical visits, size of the clinical study, and clinical sites. For analysis using test/train datasets, the splits were randomly assigned.

Blinding	CEFT was a blinded study (neither participants nor study staff will knew which treatment a participant received). PRO-ACT is a large database that aggregates anonymized clinical trial data, without disclosing explicit blinding information. The observational studies used here were not blinded to any particular therapy.

Reporting for specific materials, systems and methods

We require information from authors about some types of materials, experimental systems and methods used in many studies. Here, indicate whether each material, system or method listed is relevant to your study. If you are not sure if a list item applies to your research, read the appropriate section before selecting a response.

Materials & experimental systems

n/a	Involved in the study
☒	☐ Antibodies
☒	☐ Eukaryotic cell lines
☒	☐ Palaeontology and archaeology
☒	☐ Animals and other organisms
☒	☐ Human research participants
☒	☐ Clinical data
☒	☐ Dual use research of concern

Methods

n/a	Involved in the study
☒	☐ ChIP-seq
☒	☐ Flow cytometry
☒	☐ MRI-based neuroimaging
