# Supplementary material for: Identifying patterns in amyotrophic lateral sclerosis progression from sparse longitudinal data
Source: Nat Comput Sci. 2022 Sep 8;2(9):605–16. doi: 10.1038/s43588-022-00299-w (PMC10766562; doi:10.1038/s43588-022-00299-w)
Supplement: Supplementary file 4 — Editorial Assessment Report [file 43588_2022_299_MOESM4_ESM.pdf]

nature portfolio GUIDED OPEN ACCESS

Contents of this report

1. **Manuscript details**: overview of your manuscript and the editorial team.
2. **Review synthesis**: summary of the reviewer reports provided by the editors.
3. **Editorial recommendation**: personalized evaluation and recommendation from all 3 journals.
4. **Annotated reviewer comments**: the referee reports with comments from the editors.
5. **Open research evaluation**: advice for adhering to best reproducibility practices.

About the editorial process

Because you selected the **Nature Portfolio Guided Open Access** option, your manuscript was assessed for suitability in three of our titles publishing high-quality work in your field of research. More information about Guided Open Access can be found here.

Collaborative editorial assessment

Your editorial team discussed the manuscript to determine its suitability for the Nature Portfolio Guided OA pilot. Our assessment of your manuscript takes into account several factors, including whether the work meets the technical standard of the Nature Portfolio and whether the findings are of immediate significance to the readership of at least one of the participating journals in the Guided OA pilot.

Peer review

Experts were asked to evaluate the following aspects of your manuscript:

- **Novelty** in comparison to prior publications;
- **Likely audience** of researchers in terms of broad fields of study and size;
- **Potential impact** of the study on the immediate or wider research field;
- **Evidence** for the claims and whether additional experiments or analyses could feasibly strengthen the evidence;
- **Methodological detail** and whether the manuscript is reproducible as written;
- Appropriateness of the **literature review**.

Editorial evaluation of reviews

Your editorial team discussed the potential suitability of your manuscript for each of the participating journals. They then discussed the revisions necessary in order for the work to be published, keeping each journal's specific editorial criteria in mind.

Journals in the Nature portfolio will support authors wishing to transfer their reviews and (where reviewers agree) the reviewers' identities to journals outside of Springer Nature.
If you have any questions about review portability, please contact our editorial office at guidedoa@nature.com.

nature portfolio GUIDED OPEN ACCESS

Manuscript details

Tracking number	Submission date	Decision date	Peer review type
GUIDEDOA-21-00225	**Aug 7, 2021**	**Dec 23, 2021**	Single-blind

Manuscript title	Author details
Identifying Patterns of ALS Progression from Sparse Longitudinal Data<br><br>**Preprint:** A preprint is available at medRxiv.	Ernest Fraenkel<br><br>**Affiliation:** MIT

Editorial assessment team

**Primary editor**	**Ananya Rastogi**<br>**Home journal:** *Nature Computational Science*<br>**ORCID:** **0000-0003-3030-8535**<br>**Email:** ananya.rastogi@nature.com
**Other editors consulted**	**Christian Schnell,** *Nature Communications*, **ORCID:** 0000-0002-3499-9217<br><br>**Karli Montague-Cardoso,** *Communications Biology*, **ORCID:** 0000-0001-6614-9068
**About your primary editor**	Ananya joined Nature Portfolio in 2020 as an Associate Editor for *Nature Computational Science*. Her training and research experience during her Masters involved the use of cellular automata to study host-pathogen population dynamics. Following this, her doctorate was in the field of Systems Immunology where she worked on elucidating mechanisms of T cell mediated killing of infected cells. She has pursued research projects in various sub-fields of Computational Biology such as population genetics, modeling of intracellular dynamics and ecological modeling. Her interests include development of computational techniques to gain new insights into biological systems.

Editorial assessment and review synthesis

Editor’s summary and assessment	To model the full complexity of ALS progression, the authors propose a computational method that aggregates patient trajectories into trajectory clusters by determining the overall shape of the trajectories in each cluster using Gaussian processes, and determining the number of clusters using a Dirichlet process mixture model. To implement the model, data on longitudinal ALSFRS-R (ALS Functional Rating Scale Revised: ALSFRS-R measures 12 aspects of physical function where each function is scored from 4 (normal) to 0 (no ability)) scores was obtained from four studies. The authors compared the MoGP against two benchmark linear models: a slope model (SM), which is patient-specific, and a MoGP model with a linear kernel (LKM), which clusters patients using a simple parametric model. Across four different datasets, error in the MoGP model was lower than the LKM and SM.<br><br>Clinical data for ALS patients can often be incomplete or sparse, and the authors evaluated MoGP performance in these settings. For one dataset, PRO-ACT, when only three or six months of data are provided, the SM and LMK are the most accurate. However, when one or more years of training data were provided, the MoGP model outperformed the LKM and SM and more accurately predicted future disease progression. They also show that MoGP can be used to characterize patterns of decline using other indicators of disease progression.<br><br>While the computational framework is not necessarily novel to the computational science community (since the different steps and modules have been used before in other research works), it could potentially make a difference in the ALS field. There is an improvement in performance against SM and LMK in terms of mean error as seen in Fig. 2. Regarding performance with sparse data, SM and LMK are better with three or six months of data which was a bit of a concern since it doesn't perform the best with very sparse data.

**Editorial synthesis of reviewer reports**	Reviewer #1 has mentioned that the authors have over-represented the clinical significance of their clusters and they should mention similar use cases of MoGP in other diseases. They have also indicated the need for assessing the population-level MoGP generalizability as compared to the Gaussian models for each individual patient.<br><br>Reviewer #2 has requested technical simplification and comparing the outcome of their model previously published work.<br><br>Reviewer #3 has mentioned that the claims are overstated and more details are needed about the datasets used in the paper along with implementation on fewer data points to test the robustness.<br><br>Points to be addressed for *Nature Computational Science* (NCS): The editors at NCS will need to see addressed all points raised by the reviewers in full.<br><br>Points to be addressed for *Nature Communications*: The editors at *Nature Communications* think that the advance provided by the study is sufficient for their journal. The editors at *Nature Communication*s will need to see addressed all points raised by the reviewers in full.<br><br>Points to be addressed for *Communications Biology*: The editors at *Communications Biology* will need to see addressed all points raised by the reviewers in full.

Editorial recommendation

Nature Computational Science<br><br>Major revisions with extension of the work	The editors at NCS think that, despite the computational framework not being novel in a broad sense, the work could have a positive impact in the ALS field. They will need to see addressed all points raised by the reviewers in full. Additionally, they would like to see qualitative or quantitative discussion on how the proposed method can be applied in domains different from the ALS one.
Nature Communications<br><br>Major revisions	The editors at *Nature Communications* think that the advance provided by the study is sufficient for their journal. The editors at *Nature Communications* will need to see addressed all points raised by the reviewers in full.
Communications Biology<br><br>Major revisions	The editors at *Communications Biology* also think that the advance provided by the study is sufficient for their journal. The editors at *Communications Biology* require that all of the points raised by the reviewers are addressed as much as is feasibly possible and any caveats or limitations are clearly discussed where additional data cannot be provided.

Next steps

**Editorial recommendation 1:**	Our top recommendation is to revise and resubmit your manuscript to *Nature Computational Science.* We feel the additional experiments required are reasonable and in addition, we would like to see applications of the proposed methodology to multiple domains to establish the broad applicability of the study.
**Editorial recommendation 2:**	You may also choose to revise and resubmit your manuscript to *Nature Communications.* This option might be best if the requested experimental revisions are not possible/feasible at this time.
**Editorial recommendation 3:**	You may also choose to revise and resubmit your manuscript to *Communications Biology.*

Revision

To follow our recommendation, please upload the revised manuscript files using **the link provided in the decision letter**. Should you need assistance with our manuscript tracking system, please contact Adam Lipkin, our Nature Portfolio Guided OA support specialist, at guidedOA@nature.com.

Revision checklist

- ☐ Cover letter, stating to which journal you are submitting
- ☐ Revised manuscript
- ☐ Point-by-point response to reviews
- ☐ Updated Reporting Summary and Editorial Policy Checklist
- ☐ Supplementary materials (if applicable)

Submission elsewhere

If you choose not to follow our recommendations, you can still take the reviewer reports with you.

Option 1: Transfer to another Nature Portfolio journal
Springer Nature provides authors with the ability to transfer a manuscript within the Nature Portfolio, without the author having to upload the manuscript data again. To use this service, **please follow the transfer link provided in the decision letter.** If no link was provided, please contact guidedOA@nature.com.

> *Note that any decision to opt in to In Review at the original journal is not sent to the receiving journal on transfer. You can opt in to In Review at receiving journals that support this service by choosing to modify your manuscript on transfer.*

Option 2: Portable Peer Review option for submission to a journal outside of Nature Portfolio
If you choose to submit your revised manuscript to a journal at another publisher, we can share the reviews with another journal outside of the Nature Portfolio if requested. You will need to request that the receiving journal office contacts us at guidedOA@nature.com. We have included editorial guidance below in the reviewer reports and open research evaluation to aid in revising the manuscript for publication elsewhere.

Annotated reviewer reports

The editors have included some additional comments on specific points raised by the reviewers below, to clarify requirements for publication in the recommended journal(s). However, please note that all points should be addressed in a revision, even if an editor has not specifically commented on them.

Reviewer #1 information	
**Expertise**	Predictive Medicine; Big Data; Machine Learning; ALS; Alzheimers
**Editor's comments**	The reviewer has provided an overall positive assessment of the paper, but please see the following major comments:<br>- authors have over-represented the clinical significance of their clusters given that, unlike other biomedical MoGP models, the authors were not able to provide clear interpretability of the clusters.<br>- The authors should briefly mention similar use cases of MoGP in other diseases.<br>- the results need to be better separated to reflect the different objectives from an ALS domain standpoint.<br>- As a baseline, authors need to make a personalized guassian regression model for each patient and then assess the population-level MoGP generalizability as compared to the Gaussian models for each individual patient.<br>- The authors do not directly address why the previous models are more accurate than the present MoGP with less training data years.

Reviewer #1 comments	
**Section**	**Annotated Reviewer Comments**
**Remarks to the Author: Overall significance**	The authors present a mixture of Gaussian process (MoGP) method followed by Dirichlet modeling to predict ALS decline over time in sub-populations or "clusters" across 4 different cohorts. The overall method of time series prediction and clustering over longitudinal data, including temporal disease progression, is not novel and has been successfully performed in other diseases like Alzheimers (Peterson, et. al., NeurIps, 2018), multiple sclerosis (Zhao, et. al., 2015, IEEE Conference in Data Mining), and longitudinal omics (Cheng, et. al., 2019, Nature Communications), and many others. While the methods here are not novel, their application does further solidify the hypothesized non-linearities present in clinical ALS. Presently, the authors have over-represented the clinical significance of their clusters given that, unlike other biomedical MoGP models, the authors were not able to provide clear interpretability of the clusters. The developed model is of interest to the ALS field, although revisions are suggested to better frame the method and results in a manner that realistically portrays current significance.

1. While MoGP has not been the focus of prior ALS models, it has been used in other similar temporal disease predictions, including identifying sub-populations based on disease progression. The authors should briefly mention similar use cases in other diseases. This is important context, particularly for readers who may not have a machine learning background.

This point would be required for further consideration by NCS.

2. The results consist of 3 main aspects: prediction of ALS decline using MoGP, identifying of clusters of patients with similar progression patterns, assessing non-linearity. While these tasks are inter-related from a method standpoint, the results need to be better separated to reflect the different objectives from an ALS domain standpoint. This could be done with structural format and headings, as well as order of presentation. First discuss the ability of the model to predict a given ALS patient's progression. Then discuss the clustering. Finally, discuss the presence of linear and non-linear clusters. A sub-section of the last section would be comparing the MoGP results to the linear slope models and other linear methods of ALS prediction previously utilized in the literature.

3. Currently the authors are comparing their population MoGP model results to the linear models for individual patients (patient slope models). This makes sense for a sub-section emphasizing the importance of having a method like MoGP that is "flexible" and can model both the predominantly non-linear progressions as well as the smaller portion of linear trajectories. However, it was surprising that the authors did not include the most obvious baseline: making a personalized guassian regression model for each patient and then assess the population-level MoGP generalizability as compared to the Gaussian models for each individual patient. This would be a more apple-to-apples comparison for the sake of generalizability.

This point would be required for further consideration by NCS and *Nature Communications*, but would only have to be applied to a subset of patients for consideration at *Communications Biology*.

4. The clustering of progression patterns is certainly of clinical interest and significance. However, the clinical significance of the cluster results are over-stated. The authors do not make clear domain connections to the large numbers of clusters. The only domain content indicated by clusters was the "cliff", linear, and sigmoid hypotheses. Supplementary Table 5 indicates there is a significantly different number of clusters as a function of sample size. If more clear connections to domain features cannot be made within the scope of the present work, the authors need to simply pull back on their language and note that connecting features to the clusters would be part of future work.

5. The authors do not directly address why the previous models are more accurate than the present MoGP with less training data years. This reviewer suspects it has to do with the mixing parameter. This could be easily evaluated with a parameter sensitivity analysis. Once proven, this result would add additional credibility to the

	MoGP model presented and help provide better constraints as to what is needed (sample size, training years, visits per patient, etc.) to make the MoGP model best suited for future predictions compared to prior ALS models.<br>**This point would be required for further consideration by all three journals.**<br><br>6. Most machine learning/AI modeling papers have a model workflow or pipeline figure that clearly articulates the steps of the workflow and/or involved algorithm(s). Such a figure would really help this work. Additionally, have a pseudocode table or figure with more pertinent algorithm details in the supplement would help...particularly for training, parameter tuning, and optimization steps.<br><br>7. MINOR: The authors need to revisit the technical language. The use of first person language and pronouns throughout is more in line with an IEEE conference proceeding than a clinical or domain journal.<br>**This point was also noted by Reviewer #2.**
**Remarks to the Author: Impact**	The presented MoGP model definitely adds to present discussion in the field that ALS progression is predominantly non-linear. The overwhelming number of non-linear clusters is the most impactful result. However, the paper in its present form, does not compare to enough baselines to illustrate outright ALS progression prediction superiority across the board - in other words, it does not prove it's the best ALS model out there. The clusters are also very interesting in terms of clearly illustrating the preponderance of non-linear progressions, but they fail to fully connect to domain features that a clinical audience will appreciate. The authors do write some text with a couple of cluster examples and how they map to survival; however, more work is needed to make this a key point [if the authors want this to be a key point within their present scope of work]. In summary, focusing more on the non-linear result (which is clearly and quantitatively proven) is the strongest part of the work. However, that result is somewhat buried in the present text. Restructuring would help emphasize this finding more and minimize some of the less impactful areas where future work is still needed.
**Remarks to the Author: Strength of the claims**	1. The authors should compare the population MoGP model to single patient Gaussian regression models. This is more of an apples-to-apples comparison. The comparison to the linear slope models and such should only be used to emphasize the necessity to model non-linearity.<br><br>2. If the authors want to make the clusters be central to their work beyond illustrating the number of clusters that were non-linear progression versus linear, more detail and context needs to be given to the clusters' ties to clinical metrics. The few sentences with sparse examples on survival and respiratory function are not enough. OR the authors need to tone the language down on the significance of the clusters to only focus on importance of non-linearity and then write a limitations and future directions section to discuss future mapping of clusters to other clinical

	variables/features, citing basic examples there.<br>**Reviewer #3 also notes the need to discuss limitations. Please qualify the language and expand on potential limitations, for further consideration at all three journals.**
**Remarks to the Author: Reproducibility**	1. The authors need to provide more information on training and optimization protocols. Pseudocode tables would be helpful context.<br><br>2. While the code will be provided, some more details are necessary in the paper. Also, the authors give no detail in the paper on the types of software packages used, what type of computational environment the model was run on, etc.<br>**For the sake of reproducibility, please provide more detail on the code as requested by the reviewer, and as outlined in the Open Research Evaluation below.**

Reviewer #2 information

**Expertise**	Clinical neurophysiology; Neuromuscular disorders; Amyotrophic Lateral Sclerosis
**Editor's comments**	The reviewer has provided an overall positive assessment of the paper which focused on the application rather than the methodology. Please see the following major comments:<br><br>- Two of the datasets used are overlapping. Authors should use other large databases which are available.<br>- For this model the authors do not mention the number of required patients for its development. Exclusion criteria are very loose and arbitrary.<br>- Authors should compare the outcome of their model with the ones published applying different models.

Reviewer #2 comments

**Section**	**Annotated Reviewer Comments**
**Remarks to the Author: Overall significance**	In this study, the authors proposed a new approach to quantity disease progression in ALS. Since linear models are not ideal, the authors explored aggregating patient trajectories in individualized clusters, each with a specific course, regarding rate and curve features.<br><br>Overall, this text is not simple to be followed by most neurologists caring ALS patients. To reach greater clinical impact some technical simplification is recommended, if this is the target.<br>**Please provide sufficient context, to improve readability.**<br><br>The authors used 4 databases. Three are relatively small, Answer ALS, CEFT and EMORY, regarding the first we are not aware who introduced the data in the site (patients?), concerning the latter, the very fast rate of decline indicates that it represents a quite specific group of patients. Two databases (PRO-ACT and CEFT), they partially overlap, which is probably not a good solution regarding training and validation of their model. Other large databases are available, in particular in Europe (Westeneng HJ, et al, Prediction of personalised prognosis in patients with amyotrophic lateral sclerosis: development and validation of a prediction model, Lancet Neurology 2018), which could be used in this study.<br>**Validation on larger databases would strengthen the claims of the study and would be recommended for NCS.**<br><br>For this model the authors do not mention the number of required patients for

	its development. Exclusion criteria are very loose and arbitrary. Patients with a first visit more than 24 or 36 months after disease onset would not be accepted in a trial (the authors propose that their tool could be used in future clinical trials), they decided for 7 years; and improvement of ALSFRS-R greater 6 points is never observed in an ALS clinic (if the diagnosis is correct), why not 2 or 3, considering and acceptable fluctuation? Did they include patients with PEG or NIV at entry?<br>**Due to the focus on methodological advance at NCS, these would need to be addressed.**<br><br>Results are good. Regarding survival, it would be convenient to compare outcome of their model with the ones published applying different models (Westeneng HJ, et al, Prediction of personalised prognosis in patients with amyotrophic lateral sclerosis: development and validation of a prediction model, Lancet Neurology 2018). Some results using FVC predicted value and ALSFRs-R subscore were mentioned in results (see figures), but there no relevant information in methods about these analyses.<br>**Comparison to each of these models would be required for further consideration by NCS. Comparison to at least one model would be required for further consideration by *Nature Communications* and *Communications Biology*.**<br><br>Discussion is appreciated.<br><br>Minor Comments:<br>Last paragraph of the Introduction summarized the article, which is not necessary.<br><br>The text is somewhat repetitive in some parts, for example last paragraph on page 4 is replicated in Modelling Approach on the next page, and PRO-ACT features are described on pages 9 and 10.
**Remarks to the Author: Impact**	This is a good work, with potential great impact. Possibly *Nature Computational Science* would be the best room.<br>**While we appreciate the reviewer's input, we must emphasize that all decisions regarding publication are made solely by editors.**
**Remarks to the Author: Strength of the claims**	ALS is a very competitive area, and computational modelling is a new exciting field. After revision, this manuscript has a great chance of a relevant impact. To use another large data base in addition to PRO-ACT would strengthen their conclusions, they used 3 other relatively small, and one with overlapping with PRO-ACT.
**Remarks to the Author: Reproducibility**	I believe this could be reproduced by other authors.

Reviewer #3 information

**Expertise**	Neurology, prediction, Bayesian statistics
**Editor's comments**	The reviewer has provided an overall positive assessment of the paper, but please see the following major comments:<br><br>- Authors should tune down their claims a bit: e.g. they refer to "modelling ALS progression", but this is much broader than what they do; and other sentences in abstract and introduction as well.<br>- Authors should also evaluate the results with the sigmoidal model – although the reviewer doesn't really clarify why.<br>- Analyses with even fewer data points are needed, at least to understand how really robust the model is.<br>- More details about monotonic inductive bias are needed.<br>- Clusters of the non-PRO-ACT dataset must be presented with at least 90% of the data; in addition, computing a similarity score across the different clusters would be better for understanding how different they are.<br>- A discussion about limitations is needed.

Reviewer #3 comments

Section	Annotated Reviewer Comments
**Remarks to the Author: Overall significance**	This study provides a characterisation of the longitudinal trajectory of the ALSFRS-R in amyotrophic lateral sclerosis. The developed model was also validated in other datasets. The result is original and can be applied to other fields where longitudinal data is available and behaves non-linear.
**Remarks to the Author: Impact**	Because of the complexity of the model (which a personally appreciate and that is explained and investigated well by the authors) I have some doubts about the implementation in practice.
**Remarks to the Author: Strength of the claims**	Ramamoorthy et al. studied the longitudinal trajectory of the revised version of the amyotrophic lateral sclerosis functional rating scale (ALSFRS-R). The authors developed and validated a sophisticated Bayesian non-linear model for the longitudinal trajectory of the ALSFRS-R. Reviewing this well-performed study was a great pleasure but I have also some comments aiming to further improve this study.<br><br>1. The authors frame their study as 'modelling ALS progression'. ALS progression is, however, much broader than patients daily functioning which is measured by the ALSFRS-R. It would be great if the authors could be clearer

	about this throughout the abstract and manuscript.<br><br>2. I agree with the authors that characterizing heterogeneity in ALS is important but the last sentence of the abstract 'Our results provide a critical advance in characterizing the heterogeneity in disease progression patterns of' is somewhat overstated. This also applies to the last sentence of the introduction.<br><br>3. Minor. In paragraph 2 of the introduction, the authors discuss the change in ALSFRS-R slope that is used in clinical trials. They classify ~0.4 points difference as a small effect, but given that the average decline of the ALSFRS-R is 0.5-1.0 points per month in population-based datasets (in trial populations it might be somewhat higher) this needs to be adjusted. Moreover, edaravone is not approved in Europe.<br><br>4. Minor. In the first sentence of the fourth paragraph of the introduction a typo might have occurred ('the more a more').<br><br>5. Table 1. The distribution of 'number of visits' and 'months followed' can be very skewed. A median and range (or interquartile range) would be more appropriate. It is unclear how the ALSFRS-R slope was calculated in this table. This is important because the degree of decline can be very skewed and, if possible, a more robust measure of this slope would be preferred over the mean and standard deviation. Finally, the number of characteristics provided is too little to be sufficiently informed about the datasets used. In summary, more detail is needed.<br><br>6. Around 3000 participants from the PRO-ACT database were included (which was by far the largest dataset used). Can the authors please comment on this selection? Which criteria were used to select these patients from the PRO-ACT database and why? What happened when more patients from the PRO-ACT database were included?<br><br>7. Subjects with at least 4 visits were used for prediction and subjects with at least 10 visits were used for assessing interpolation. Could the authors please provide analyses of what happened when fewer data points were available (to really demonstrate how robust each model is to sparse data)? This is even more important because (even from a trial population with usually a lot of measurements, i.e. PRO-ACT) >50% of the subjects were excluded because they have less than the data points needed. And could the authors please evaluate not only the MoGP, SM and LKM model but also the sigmoidal model? And which results were obtained when performing these analyses in the other datasets?<br>**This point would be necessary for further consideration at NCS or *Nature Communications*.**

	8. When reading the methods section about 'Model Generalizability' I first interpreted that the model developed in the PRO-ACT database (i.e. reference model) was modified before it was applied to other datasets. After reading the results it became clear that this was not the case. Could the authors please clarify that the primary analysis was to develop a model in the PRO-ACT database and apply this unchanged to the other datasets? The 'study specific models' can be mentioned as additional sensitivity analyses to investigate possible overfitting of the reference model. And as a minor comment, it could be added that the test and train datasets were split randomly.<br><br>9. From the methods it was somewhat unclear how the 'monotonic inductive bias' was incorporated. After reading the supplement this became clear for the 'negative linear mean function', but I still have some difficulties with interpreting the 'threshold function'. Could the authors please consider improving the section about 'monotonic inducive bias'?<br><br>10. The authors claim: 'The heterogeneity of the populations enabled us to measure the robustness of our model to data collection methods and the generalizability of ALS progression patterns between varying study populations'. This claim about heterogeneity is very much dependent on the underlying causes of this heterogeneity and not so much on the few measures provided (ALSFRS-R slope and follow-up duration). This refers also to my comment about table 1.<br><br>11. In supplementary figure 1 different clusters of the non-PRO-ACT dataset are plotted. These figures, however, display only a relatively small part of the data: AALS 284 of 456 patients (62%), CEFT 216 of 476 (45%) and EMORY 282 of 399 (71%). Could the authors please show an increased number of figures to demonstrate the different clusters? I would suggest that clusters of at least 90% of the data would be provided. This also applies to the clusters found in the PRO-ACT database (figure 1) which includes 1573 out of 2923 patients (54%). If Figure 1 becomes too large it possibly can be provided supplementary. Moreover, different clusters look very similar. Can the authors please provide a similarity score between clusters?<br><br>12. 92 clusters were found using the PRO-ACT data, while in the other datasets a maximum of 34 clusters was found. This is intrinsically related to the methods used but the meaning of this difference needs to be discussed in the discussion.<br><br>13. Minor. Based on the text, the 'greater than sign' in supplementary tables 2, 3 and 4 should be replaced by a 'greater than or equal to sign'.<br><br>14. Minor. I think that figure 2a and 2b have different messages and it might result in interpretation difficulties to combine them. Maybe these figures can be split up into separate figures?

	15. Figure 3. These figures are now somewhat difficult to read, especially the error bars (which are very small). I think that it would be much more straightforward to interpret when the authors plot the full distribution of the (absolute) deviation of points from the modelled mean (if needed with a log or square root transformation). Some readers might for example interpret the first blue bar in Figure 3A as an error of 3 points (with very small error bars) which could be interpreted by some readers as that the model has nearly always an error of 3 points, which is a lot. Significant differences with other models have only a very limited meaning because all these models perform suboptimally. This interpretation can be prevented by just plotting the absolute differences and it provides also more insight into the full distributions of errors.<br><br>16. Minor. In the supplement, the authors describe the ∝ parameter, which indicates the scaling parameter of the beta prior, but no value for this parameter is provided.<br><br>17. I could not find a discussion of possible limitations. Besides my suggestions above, I think that the lack of population-based datasets can be seen as a potential limitation and could lead to selection bias. Furthermore, attrition bias is a common problem in ALS research. Could the authors please discuss these two biases that could be present in their study and what the meaning of these biases is for the interpretation of their study?<br>**This point was also noted by Reviewer #1. Please qualify the language and expand on potential limitations, for further consideration at all three journals.**
**Remarks to the Author: Reproducibility**	The analyses were appropriate. The developed model was validated in other datasets. The code for this study is provided online.

Data availability
**Data availability statement**
Thank you for including a Data Availability statement. However, we noted that you have only indicated that data are available upon request. The data availability statement must make the conditions of access to the “minimum dataset” that are necessary to interpret, verify and extend the research in the article, transparent to readers.<br><br>In addition, Nature Portfolio policies include a strong preference for research data to be archived in public repositories. For data types without specific repositories, we recommend that data are deposited in a generalist repository such as figshare or Dryad. More information about our data availability policy can be found here:<br>https://www.nature.com/nature-portfolio/editorial-policies/reporting-standards#availability-of-data<br><br>See here for more information about formatting your Data Availability Statement:<br>http://www.springernature.com/gp/authors/research-data-policy/data-availability-statements/12330880
**Mandatory data deposition**
For your RNA sequencing data, submission to a community-endorsed, public repository is mandatory for publication in a Nature Portfolio journal and is best practice for publication in any venue. Accession numbers must be provided in the paper. Examples of appropriate public repositories are listed below:<br><br>-Gene Expression Omnibus (Microarray or RNA sequencing data)<br>-Sequence Read Archive (high-throughput sequence data)<br>-The European Nucleotide Archive (ENA)
For your genome-wide association study, submission of the full linked genotype dataset to a community-endorsed, public repository is mandatory for publication in a Nature Portfolio journal and is best practice for publication in any venue. Accession numbers must be provided in the paper.<br><br>For this data type, we recommend submission to the NCBI Sequence Read Archive (SRA):<br>https://www.ncbi.nlm.nih.gov/sra<br><br>We also strongly encourage you to deposit full summary statistics and other related data to a generalist repository, such as figshare or Dryad. However, it may be acceptable to include the summary statistics in the supplementary information.
More information on mandatory data deposition policies at the Nature Portfolio can be found at<br>http://www.nature.com/authors/policies/availability.html#data

Please visit https://www.springernature.com/gp/authors/research-data-policy/repositories/12327124 for a list of approved repositories for each mandatory data type.
**Other data requests**
All source data underlying the graphs and charts presented in the main figures must be made available as Supplementary Data (in Excel or text format) or via a generalist repository (eg, Figshare or Dryad). This is mandatory for publication in a Nature Portfolio journal, but is also best practice for publication in any venue.<br><br>The following figures require associated source data: Figures 1 to 6.
Springer Nature strongly supports data sharing and believes that all datasets on which the conclusions of the paper rely should be available to readers. We encourage authors to ensure that their datasets are either deposited in publicly available repositories (where available and appropriate) or presented in the main manuscript or additional supporting files whenever possible.<br><br>Please see Springer Nature's information on recommended repositories:<br>https://www.springernature.com/gp/authors/research-data-policy/repositories/12327124
**Data citation**
Please cite (within the main reference list) any datasets stored in external repositories that are mentioned within their manuscript. For previously published datasets, we ask that you cite both the related research article(s) and the datasets themselves. For more information on how to cite datasets in submitted manuscripts, please see our data availability statements and data citations policy:<br>https://www.nature.com/documents/nr-data-availability-statements-data-citations.pdf<br><br>Citing and referencing data in publications supports reproducible research, by increasing the transparency and provenance tracking of data generated or analysed during research. Citing data formally in reference lists also helps facilitate the tracking of data reuse and may help assign credit for individuals' contributions to research. A number of Springer Nature imprints are signatories of the Joint Declaration on Data Citation Principles, which stress the importance of data resources in scientific communication.
**Code availability and citation**
Please include a statement under the heading "Code Availability", indicating whether and how the custom code/software reported in your study can be accessed, including any restrictions to access. This section should also include information on the versions of any software used, if relevant, and any specific variables or parameters used to generate, test, or process the current dataset. Code availability statements should be provided as a separate section after the Data Availability section.<br><br>Upon publication, Nature Portfolio journals consider it best practice to release custom computer code in a way that allows readers to repeat the published results. Code should be deposited in a DOI-minting repository such as Zenodo, Gigantum or Code Ocean and cited in the reference list following the

guidelines described in our policy pages (see link below). Authors are encouraged to manage subsequent code versions and to use a license approved by the open source initiative. Full details about how the code can be accessed and any restrictions must be described in the Code Availability statement.<br><br>See here for more information about our code availability policies: https://www.nature.com/nature-portfolio/editorial-policies/reporting-standards#availability-of-computer-code<br><br>We also provide a Code and Software submission checklist that you may find useful: https://www.nature.com/documents/nr-software-policy.pdf<br><br>Please note: because of advanced features used in this form, you must use Adobe Reader to open the documents and fill it out.
**Ethics**
Please provide a 'Competing interests' statement using one of the following standard sentences:<br><br>1. The authors declare the following competing interests: [specify competing interests]<br>2. The authors declare no competing interests.<br><br>See our competing interests policy for further information: https://www.nature.com/nature-research/editorial-policies/competing-interests
Because your study includes human participants, confirmation that all relevant ethical regulations were followed is needed, and that informed consent was obtained. This must be stated in the Methods section, including the name of the board and institution that approved the study protocol.
**Reporting & reproducibility**
Nature Portfolio journals allow unlimited space for Methods. The Methods must contain sufficient detail such that the work could be repeated. It is preferable that all key methods be included in the main manuscript, rather than in the Supplementary Information. Please avoid use of “as described previously” or similar, and instead detail the specific methods used with appropriate attribution.
We encourage you to share your step-by-step experimental protocols on a protocol sharing platform of their choice. The Nature Portfolio’s Protocol Exchange is a free-to-use and open resource for protocols; protocols deposited in Protocol Exchange are citable and can be linked from the published article. More details can be found at www.nature.com/protocolexchange/about
**Statistics and data presentation**
To improve reproducibility of your analyses, please provide details regarding your treatment of outliers.

When choosing a color scheme please consider how it will display in black and white (if printed), and to users with color blindness. Please consider distinguishing data series using line patterns rather than colors, or using optimized color palettes such as those found at https://www.nature.com/articles/nmeth.1618.<br><br>The use of colored axes and labels should be avoided. Please avoid the use of red/green color contrasts, as these may be difficult to interpret for colorblind readers.
The quality of some of the figures appears to be quite low. If possible, we suggest replacing these with higher-resolution images.
**Language editing**
The English language in your text would benefit from improvement for clarity and readability. We recommend that you either ask a colleague with strong English language skills to review your manuscript or that you use one of the many English language editing services available. Two such services are provided by our affiliates:<br>● Springer Nature Editing Service: https://secure.authorservices.springernature.com/en/researcher/submit/upload<br>● American Journal Experts: https://www.aje.com/go/natureresearch/
**Other notes**
We have included as an attachment to the decision letter a version of your Reporting Summary with a few notes. This is mainly for your information, but we hope it is helpful when preparing your revised manuscript. If you decide to resubmit the manuscript for further consideration, please be sure to include an updated Reporting Summary.
